# Percutaneous kyphoplasty for a “centenarian”: A case report and a summary of technical points

**DOI:** 10.1016/j.amsu.2019.07.007

**Published:** 2019-07-09

**Authors:** Huilin Yang, Jun Ge, Hao Liu, Jun Zou

**Affiliations:** Department of Orthopaedic Surgery, The First Affiliated Hospital of Soochow University, China

**Keywords:** Percutaneous kyphoplasty, Centenarian, Diagnosis, Technology

## Abstract

**Objective:**

To present a case of 97-year-old patient performed by Percutaneous kyphoplasty (PKP) with the key technology of diagnosis and therapy.

**Methods:**

To review literatures about super-aged patients suffering from osteoporotic vertebral compression fracture and summarize the treatment procedures and technical points.

**Results:**

The centenarian patient received a satisfied follow-up efficacy using the diagnosis and key technology of PKP.

**Conclusions:**

PKP is an effective surgical procedure for the treatment of super-aged patient using the diagnosis and key technology.

## Highlights

• This work describes using Percutaneous kyphoplasty (PKP) for a “centenarian” for the very first time.
• Whereby, we discuss and summarize the technique points of PKP.
• This work summarize and discuss the treatment of vertebral compression fractures for super-aged patients.

## 1 Introduction

Osteoporotic vertebral compression fracture (OVCF) patients often suffer from severe osteoporosis and multiple vertebral compression fractures, accompanied by hypertension, diabetes, coronary heart disease, pneumonia and other underlying diseases, which result in some difficulties for the diagnosis and treatment of this disease. Conservative therapy with traditional bed rest will lead to further loss of bone mass and deterioration of underlying diseases, as well as higher mortality. Surgical treatment of elderly patients also increases the risks associated with surgery and anaesthesia, and requires careful preoperative evaluation, rational treatment regimens, highly skilled handling techniques and standardized postoperative rehabilitation exercise and care. During the treatment of elderly patients with OVCF, it can be difficult for many physicians to develop an effective treatment program, resulting in poor efficacy and poorer outcomes. This case study presents an OVCF “centenarian” patient. The “painful vertebral body” diagnosis and treatment technology we used for treatment with this case. After treatment with percutaneous kyphoplasty (PKP) key technology, satisfactory results were obtained, which provide a clinical reference for OVCF therapy. This work complies with the Surgical Case Report (SCARE) guidelines [1].

## 2 Presentation of case

**Fig. 1:**
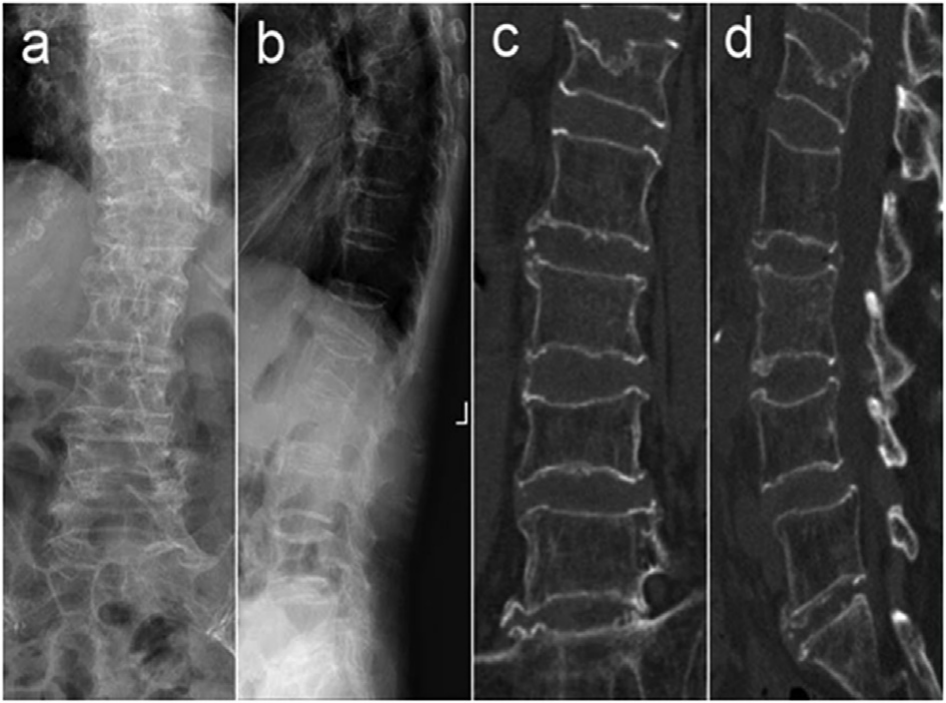
Preoperative X-ray (a, b), and crown-lost CT reconstruction (c, d), suggest T7, T12, L1 multiple vertebral compression fractures.

**Fig. 2:**
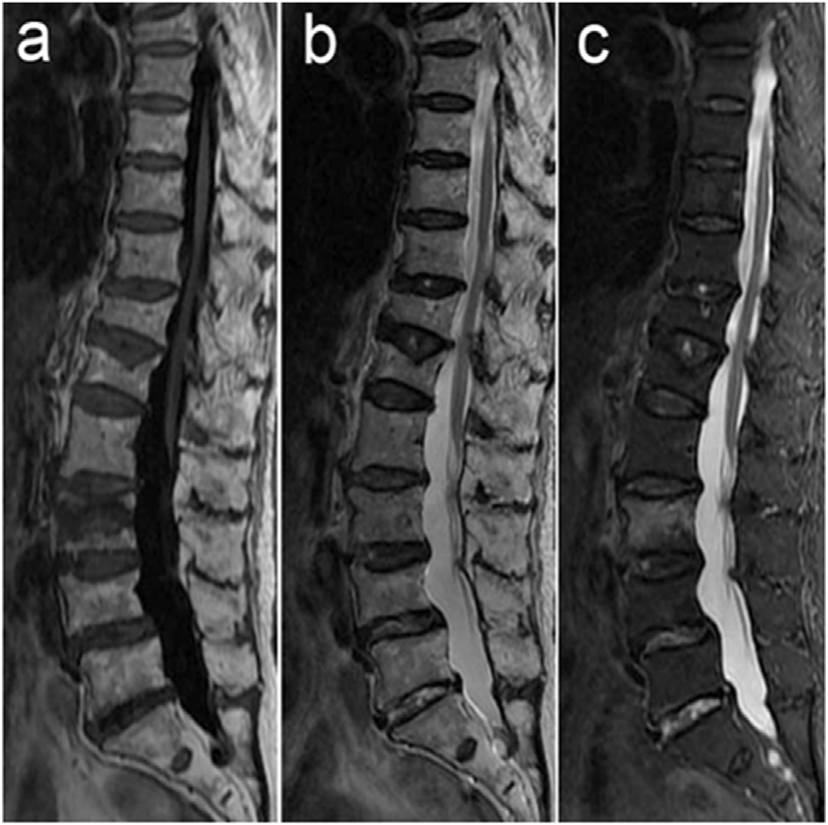
Preoperative MRI examination. L3 vertebral body showed low signal (a), T2 and other signals (b) and STIR images showed a high signal (c). This confirmed L3 as the vertebral body responsible for the pain.

**Fig. 3:**
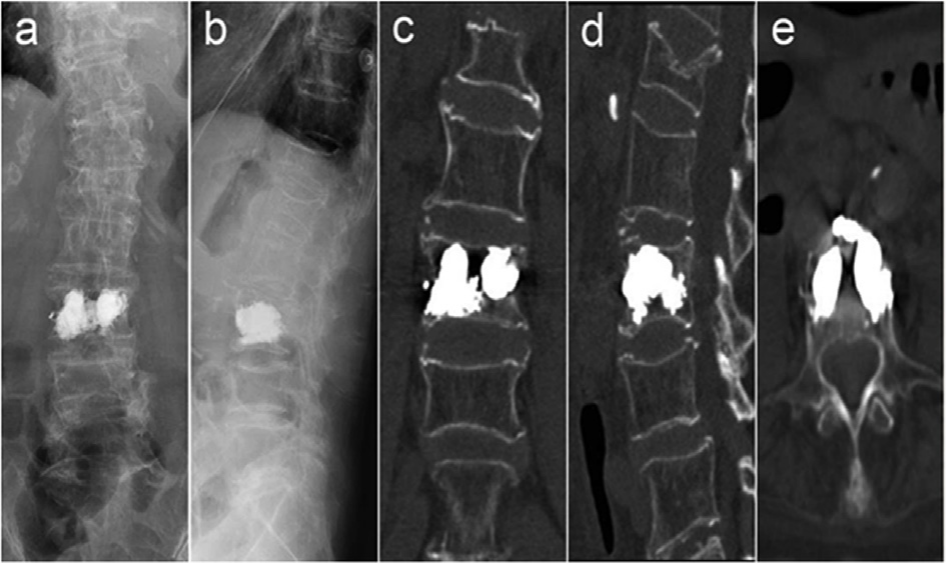
Postoperative X-ray (a, b) and crown loss, cross-sectional CT reconstruction (c, d, e) suggest that bone cement dispersion in the L3 vertebral body is good, without obvious bone cement leakage.

A 97-year-old woman was admitted to our hospital complaining of “back pain after coughing for 1 week”. There was no history of high blood pressure or diabetes, and she was mostly responsible for herself-care. After admission, radiography and CT examination results revealed multiple vertebral compression fractures at T7, T12, and L1, and osteoporosis (Fig. 1), and an average lumbar spine bone mineral density T value of −4.8. However, physical examination showed a positive percussion pain response only in the L3 vertebral spinous process, with no pain found in the rest of the vertebral segment. To further determine the patient's lower back pain source, magnetic resonance imaging (MRI) was performed, which indicated significant signal changes observable only in the Short TI Inversion Recovery (STIR) sequence of the L3 vertebral body, with the rest of the vertebral body marked by older fractures, confirming that the L3 vertebral body was the source of the pain. (Fig. 2) Therefore, L3 posterior kyphoplasty was performed under general anaesthesia, and anti-osteoporosis medication was administered pre- and post-surgery. Postoperatively, this patient's lower back pain was relieved, and she was able to resume her usual activities. Three years after surgery, the now 100-year-old patient returned to our hospital for review. There had been no recurrence of lower back pain, vertebral height had not been lost following L3 surgery, and there had been no recurring fractures within the adjacent vertebral body (Fig. 3).

## 3 Discussion

It is reported that most OVCF patients (>75%) have only minor injuries (such as coughing and sneezing) or no clear trauma. A number of patients (<25%) have suffered from falling or from more severe physical trauma. For OVCF patients, conservative treatment is most often applied, including bed rest, and local pain relief. However, more than 2 weeks of bed restraint can lead to pressure lesions for >70% of patients over 70 years of age, in addition to pulmonary infection. Furthermore, research indicates that the rate of occurrence of deep venous thrombosis in bed-rest patients is 61%, the pulmonary embolism rate ranges from 2% to 12%, lung function decreases between 25% and 50%, and the 4-year mortality rate is as high as 49.4% [2,3]. Therefore, surgical treatment to enable patients to leave their beds earlier, with rapid pain relief and to improve quality of life as soon as possible, has been one of the important goals for the treatment of elderly OVCF patients. PKP, with its advantages of reduced trauma, obvious analgesic effect, and faster postoperative rehabilitation, has been accepted by most doctors and patients [4,5]. However, elderly patients with OVCF often present with multiple vertebral fractures, severe compression fractures, vertebral wall damage and multiple comorbidities. Moreover, patients and their families worry about surgery and high risks with anaesthesia, poor surgical tolerance and greater complications for the elderly, so they do not always take the initiative in accepting surgical treatment, which often worsens the condition; on the other hand, patients in a long-term prone position have a greater need for surgery, but severe vertebral osteoporosis and peripheral wall damage increase bone cement leakage and pulmonary embolism risk, making the surgeons more reluctant to perform surgery. Therefore, physicians are often confronted with a dilemma in the treatment of elderly patients with OVCF.

In this case, the patient was 97 years old with multiple vertebral compression fractures, but without every compressed vertebral body requiring surgical treatment. We used the “painful vertebral body” [6] concept to determine which vertebral segments required surgery. Through the obvious knock tenderness within the L3 vertebral spinous process and the characteristics of MRI signal changes concerning the L3 vertebral body, we determined L3 as the pain site. PKP surgery was performed on the L3 body, without involving preventive or excessive treatment, and ensured a positive surgical outcome while avoiding unnecessary surgical trauma and risk. Elderly patients are often accompanied by scoliosis and severe osteoporosis, resulting in intraoperative fluoroscopic signs that create a lack of clarity and difficulties for an intraoperative needle and puncture operation, so we propose using a “line shadow” benchmark positioning and body surface puncture technology. This technology reduces the number of repeated intraoperative fluoroscopies, standardizes the operation, and reduces the operation time, so reducing the risks associated with long-term surgery for elderly patients. For elderly patients with OVCF, severe compression, or peripheral wall damage, we use the incremental temperature cement delivery technique [[6], [7], [8]]. We first inject small doses of early stage bone cement to hold the vertebral body wall and the anteroposterior wall of the rupture or defect area, while considering the in vivo situation and operating room temperature differences. Then, we draw late stage bone cement into the vertebral body once the previously introduced cement is close to solidification, so that bone cement and the vertebral anchor are firmly fixed, effectively reducing the risk of bone cement leakage in elderly patients.

## 4 Conclusion

This case shows that, during the diagnosis and treatment process for elderly OVCF patients, there needs to be better medical understanding of options in dealing with the vertebral body, to avoid misdiagnosis and poorly directed therapy. The minimally invasive PKP technology should be applied to achieve better outcomes for patients, specifically to reduce the risk of bone cement leakage and mortality, and to complete operations in a shorter timeframe, so that elderly patients recover more quickly and return as soon as possible to normal life. Even “centenarians” can tolerate surgery using this approach, and satisfactory results can be obtained.

## Conflicts of interest

The authors declared no conflicts of interest.

## Consent

Written informed consent was obtained from the patient for publication of this case report and accompanying images. A copy of the written consent is available for review by the Editor-in-Chief of this journal on request.

## Sources of funding

None declared sources of funding.

## Ethical approval

No ethical approval is need in case report.

## Author contribution

HL Yang: study concept or design, data collection, guide the writing of the paper.

J Ge: data collection, data analysis or interpretation, writing the paper.

H Liu: data analysis or interpretation, writing the paper.

J Zou: study concept or design, data collection, data analysis or interpretation.

## Registration of research studies

No registration of research studies is need for a case report.

## Guarantor

Dr. Huilin Yang and Dr. Jun Zou.

## Provenance and peer review

Not commissioned, internally peer reviewed.
